# Café com Reflexões Acadêmicas

**DOI:** 10.36660/abc.20250739

**Published:** 2026-03-11

**Authors:** Paolo Blanco Villela

**Affiliations:** 1 Universidade Federal do Rio de Janeiro Rio de Janeiro RJ Brasil Universidade Federal do Rio de Janeiro, Rio de Janeiro, RJ – Brasil; 2 Clínica São Vicente Rio de Janeiro RJ Brasil Clínica São Vicente, Rio de Janeiro, RJ – Brasil

**Keywords:** Café, Cafeína, Doença Arterial Coronariana, Doenças Cardiovasculares, Paraxantina

O café é uma das bebidas mais consumidas no mundo,^1^ o que, por conseguinte, torna seu principal componente — a cafeína, a substância psicoativa mais consumida globalmente.^2^ Embora existam outras fontes de cafeína, como chás, energéticos, erva-mate e refrigerantes, a principal fonte de cafeína em adultos é o café, podendo representar até 65% da ingestão diária total em determinadas populações.^2,3^

Apesar da cafeína despertar, literalmente, a atenção acadêmica por seus efeitos em diversos órgãos e sistemas, o café contém inúmeros outros compostos biologicamente ativos, que podem inclusive sofrer modificações ao longo do processo entre o grão e a xícara do consumidor.^4^ Desta forma, polifenóis, trigonelina, e mais outras centenas de substâncias presentes no café^4^ podem assumir papel coadjuvante e não desprezível, nos efeitos sistêmicos, e devem ser consideradas nas discussões sobre o tema.

Após a absorção da cafeína, segue à sua metabolização hepática através da enzima CYP1A2 do citocromo P-450, formando majoritariamente o metabólito paraxantina, mas também teobromina e teofilina em menores proporções.^5^ Embora exista variação na atividade enzimática, a relação entre a atividade e os efeitos clínicos parece ser não linear. Estudo prospectivo realizado utilizando dados do UK Biobank não encontrou evidências de que o metabolismo lento ou rápido da cafeína modificasse a associação (inversa) entre o consumo de café e a mortalidade por todas as causas.^6^ Esse resultado enfatiza a complexidade dos efeitos do café no organismo, além do fato de que diferenças genéticas interpopulacionais e outros compostos bioativos do café podem exercer efeitos paralelos.

Em relação aos efeitos no sistema cardiovascular, apesar da preocupação histórica de que o café seria prejudicial, em revisão feita por Dewland et al., os dados sugerem que o consumo moderado de café está associado a um risco reduzido de doenças cardiovasculares, incluindo hipertensão arterial, infarto do miocárdio e mortalidade total.^1^ Em concordância, Shirai et al, demostraram relação inversa entre o consumo de café e a mortalidade por doenças isquêmicas do coração em série temporal de 1990 a 2018.^7^ Cabe destacar que a definição de "moderado" é demasiadamente ampla e subjetiva e, evidentemente, os efeitos no sistema cardiovascular dependem da dose, do modo de preparo e da carga genética dos indivíduos consumidores.

Diante do exposto, do ponto de vista acadêmico, estudar esta relação não é tarefa simples. A utilização metodológica do consumo autorreferido de café e de cafeína nas pesquisas é propensa a vieses importantes.^8^ A associação habitual e social entre o consumo de café por indivíduos tabagistas, por exemplo, pode superestimar o risco cardiovascular.^9^ Além disso, por ser uma informação subjetiva, está sujeita a imprecisões na lembrança das doses e da frequência, o que leva à super ou subestimação dos dados, a depender da população questionada.

Portanto, utilizar uma régua subjetiva para estudar esta relação, pode não ser adequado em alguns cenários. Assim, estudos que avaliam a exposição a cafeína de forma objetiva tornam-se metodologicamente interessantes,^8,10^ embora com as dificuldades inerentes aos custos para a sua realização.

Essa edição dos Arquivos Brasileiros de Cardiologia, traz a publicação do estudo de Li et al., que analisou dados do National Health and Nutrition Examination Survey (NHANES) entre 2009 e 2014 e, através da avaliação de metabólitos urinários, fornece importante resultado sobre a relação da cafeína com a doença arterial coronariana (DAC).^11^ Trata-se de um estudo transversal, com 5227 registros de pacientes, onde houve cuidado no tratamento estatístico e mensuração objetiva da exposição à cafeína, destacando a importância do estudo e os resultados encontrados.^11^

Segundo os autores, níveis mais elevados de paraxantina urinária estiveram associados a menor risco de DAC, enquanto concentrações elevadas de cafeína urinária mostraram associação positiva com a incidência de DAC, especialmente entre indivíduos mexicano-americanos.^11^ Possíveis polimorfismos genéticos no perfil de metabolizadores, fatores relacionados à etnia, e a baixa prevalência de doença coronária na população estudada, também devem ser considerados na análise dos resultados.

Apesar de algumas limitações para explicar a relação causal com uma doença tempo-sensível, como a doença coronariana, e considerando o fato de, em transversalidade, a dosagem de metabolitos urinários estar sujeita à variações temporais entre o consumo-avaliação, estes dados assumem importância em cenários complexos como o que se apresenta, e devem ser considerados de maneira ampla, no conjunto de relações complexas entre o café e o organismo humano.

Desta forma, considerando as reflexões mencionadas, o estudo de Li et al.^11^ traz importante contribuição para o entendimento dos efeitos a médio-longo prazo do consumo de cafeína e a DAC. Além disso, seus resultados sugerem a necessidade de estudos prospectivos com aferições objetivas do consumo de café, em que pese sua viabilidade econômica e sua execução prática, para melhor estabelecimento de relações causais entre a bebida e seus efeitos no sistema cardiovascular.

## References

[1] 1 Dewland TA, van Dam RM, Marcus GM. Coffee and Cardiovascular Disease. Eur Heart J. 2025;46(36):3546-54. doi: 10.1093/eurheartj/ehaf421. 10.1093/eurheartj/ehaf421 40626862

[2] 2 van Dam RM, Hu FB, Willett WC. Coffee, Caffeine, and Health. N Engl J Med. 2020;383(4):369-78. doi: 10.1056/NEJMra1816604. 10.1056/NEJMra1816604 32706535

[3] 3 Drewnowski A, Rehm CD. Sources of Caffeine in Diets of US Children and Adults: Trends by Beverage Type and Purchase Location. Nutrients. 2016;8(3):154. doi: 10.3390/nu8030154. 10.3390/nu8030154 PMC4808882 26978391

[4] 4 Ludwig IA, Clifford MN, Lean ME, Ashihara H, Crozier A. Coffee: Biochemistry and Potential Impact on Health. Food Funct. 2014;5(8):1695-717. doi: 10.1039/c4fo00042k. 10.1039/c4fo00042k 24671262

[5] 5 Guessous I, Pruijm M, Ponte B, Ackermann D, Ehret G, Ansermot N, et al. Associations of Ambulatory Blood Pressure with Urinary Caffeine and Caffeine Metabolite Excretions. Hypertension. 2015;65(3):691-6. doi: 10.1161/HYPERTENSIONAHA.114.04512. 10.1161/HYPERTENSIONAHA.114.04512 25489060

[6] 6 Loftfield E, Cornelis MC, Caporaso N, Yu K, Sinha R, Freedman N. Association of Coffee Drinking with Mortality by Genetic Variation in Caffeine Metabolism: Findings from the UK Biobank. JAMA Intern Med. 2018;178(8):1086-97. doi: 10.1001/jamainternmed.2018.2425. 10.1001/jamainternmed.2018.2425 PMC6143111 29971434

[7] 7 Shirai Y, Imai T, Sezaki A, Miyamoto K, Kawase F, Abe C, et al. Change in the Association between Coffee Intake and Ischemic Heart Disease in an International Ecological Study from 1990 to 2018. Sci Rep. 2022;12(1):11319. doi: 10.1038/s41598-022-15611-x. 10.1038/s41598-022-15611-x PMC9256668 35790762

[8] 8 Laaboub N, Ranjbar S, Strippoli MF, Marques-Vidal P, Estoppey-Younes S, Ponte B, et al. Self-Reported Caffeine Consumption Miss-Matched Consumption Measured by Plasma Levels of Caffeine and Its Metabolites: Results from Two Population-Based Studies. Eur J Nutr. 2024;63(5):1555-64. doi: 10.1007/s00394-024-03351-9. 10.1007/s00394-024-03351-9 PMC11329688 38703227

[9] 9 Ding M, Bhupathiraju SN, Satija A, van Dam RM, Hu FB. Long-Term Coffee Consumption and Risk of Cardiovascular Disease: A Systematic Review and a Dose-Response Meta-Analysis of Prospective Cohort Studies. Circulation. 2014;129(6):643-59. doi: 10.1161/CIRCULATIONAHA.113.005925. 10.1161/CIRCULATIONAHA.113.005925 PMC3945962 24201300

[10] 10.Weng Z, Xu C, Xu J, Jiang Z, Liu Q, Liang J, et al. Association of Urinary Caffeine and Caffeine Metabolites with Cardiovascular Disease Risk in Adults. Nutrition. 2021;84:111121. doi: 10.1016/j.nut.2020.111121. 10.1016/j.nut.2020.111121 33515809

[11] 11 Li Q, Ke G, Liu M, Chen Y, Luo Y, Qiu L. Study on Urinary Caffeine and Caffeine Metabolite Levels in American Adults with Coronary Heart Disease: A Cross-Sectional NHANES Study (2009-2014). Arq Bras Cardiol. 2025;122(9):e20240425. doi: 10.36660/abc.20240425. 10.36660/abc.20240425 PMC12217927 41379169

