# Supplementary material for: Café com Reflexões Acadêmicas
Source: Arq Bras Cardiol. 2026 Mar 11;122(9):e20250739. [Article in Portuguese] doi: 10.36660/abc.20250739 (PMC13155786; doi:10.36660/abc.20250739)
Supplement: Translation

# Translation: Coffee with Academic Reflections

**DOI:** 10.36660/abc.20250739i

**Authors:** Paolo Blanco Villela

**Affiliations:** 1 Rio de Janeiro RJ Brazil Universidade Federal do Rio de Janeiro, Rio de Janeiro, RJ – Brazil; 2 Rio de Janeiro RJ Brazil Clínica São Vicente, Rio de Janeiro, RJ – Brazil

**Keywords:** Coffee, Caffeine, Coronary Artery Disease, Cardiovascular Diseases, Paraxanthine

Coffee is one of the most consumed beverages in the world,^1^ which consequently makes its main component — caffeine — the most consumed psychoactive substance globally.^2^ Although there are other sources of caffeine, such as teas, energy drinks, yerba mate, and soft drinks, the main source of caffeine in adults is coffee, which can represent up to 65% of the total daily intake in certain populations.^2,3^

Although caffeine literally attracts academic attention for its effects on various organs and systems, coffee contains numerous other biologically active compounds, which can even undergo modifications during the process between the bean and the consumer's cup.^4^ Thus, polyphenols, trigonelline, and hundreds of other substances present in coffee^4^ can play a supporting and not insignificant role in systemic effects, and should be considered in discussions on the subject.

Following absorption, caffeine undergoes hepatic metabolism via the cytochrome P-450 enzyme CYP1A2, primarily forming the metabolite paraxanthine, but also theobromine and theophylline in smaller proportions.^5^ Although there is variation in enzymatic activity, the relationship between activity and clinical effects appears to be non-linear. A prospective study using data from the UK Biobank found no evidence that slow or fast caffeine metabolism modified the (inverse) association between coffee consumption and all-cause mortality.^6^ This result emphasizes the complexity of coffee's effects on the body, in addition to the fact that interpopulation genetic differences and other bioactive compounds in coffee may exert parallel effects.

Regarding the effects on the cardiovascular system, despite historical concerns that coffee is harmful, a review by Dewland et al. suggests that moderate coffee consumption is associated with a reduced risk of cardiovascular disease, including hypertension, myocardial infarction, and total mortality.^1^ In agreement, Shirai et al. demonstrated an inverse relationship between coffee consumption and mortality from ischemic heart disease in a time series from 1990 to 2018.^7^ It is important to highlight that the definition of "moderate" is excessively broad and subjective, and evidently, the effects on the cardiovascular system depend on the dose, the method of preparation, and the genetic makeup of the individual consumers.

Given the above, from an academic point of view, studying this relationship is not a simple task. The methodological use of self-reported coffee and caffeine consumption in research is prone to significant biases.^8^ The habitual and social association between coffee consumption by smokers, for example, may overestimate cardiovascular risk.^9^ Furthermore, because it is subjective information, it is subject to inaccuracies in recalling doses and frequency, which leads to over- or underestimation of data, depending on the population surveyed.

Therefore, using a subjective scale to study this relationship may not be appropriate in some scenarios. Thus, studies that objectively assess caffeine exposure become methodologically interesting,^8,10^ although with the inherent difficulties and costs involved in conducting them.

This edition of the *Arquivos Brasileiros de Cardiologia* features the publication of the study by Li et al., which analyzed data from the National Health and Nutrition Examination Survey (NHANES) between 2009 and 2014 and, through the evaluation of urinary metabolites, provides important results on the relationship between caffeine and coronary artery disease (CAD).^11^ This is a cross-sectional study, with 5227 patient records, where care was taken in the statistical treatment and objective measurement of caffeine exposure, highlighting the importance of the study and the results found.^11^

According to the authors, higher levels of urinary paraxanthine were associated with a lower risk of CAD, while elevated urinary caffeine concentrations showed a positive association with the incidence of CAD, especially among Mexican-American individuals.^11^ Possible genetic polymorphisms in the metabolizer profile, factors related to ethnicity, and the low prevalence of coronary heart disease in the studied population should also be considered in the analysis of the results.

Despite some limitations in explaining the causal relationship with a time-sensitive disease, such as coronary heart disease, and because, in cross-sectional studies, the measurement of urinary metabolites is subject to temporal variations between consumption and evaluation, these data are important in complex scenarios such as the one presented, and should be considered broadly within the complex set of relationships between coffee and the human body.

Thus, considering the aforementioned reflections, the study by Li et al.^11^ makes an important contribution to understanding the medium- to long-term effects of caffeine consumption and CAD. Furthermore, their results suggest the need for prospective studies with objective measurements of coffee consumption, despite their economic viability and practical implementation, to better establish causal relationships between the beverage and its effects on the cardiovascular system.
